# HPV vaccination uptake and administration from 2006 to 2016 in a commercially insured population of the United States

**DOI:** 10.1186/s12889-021-11664-1

**Published:** 2021-09-06

**Authors:** Vimalanand S. Prabhu, Neha Bansal, Zhiwen Liu, Rodney Finalle, Martin Sénécal, Smita Kothari, Kemar Trowers, Evan Myers

**Affiliations:** 1 Center for Observational and Real-World Evidence, Merck Sharp & Dohme Corp., a subsidiary of Merck & Co., Inc, 2000 Galloping Hill Rd, Kenilworth, NJ 07033 USA; 2 Complete HEOR Solutions (CHEORS), North Wales, PA USA; 3 Division of Women’s Community and Population Health, Department of Obstetrics & Gynaecology, Duke University Medical Center, Durham, NC USA

**Keywords:** Human papillomavirus, HPV vaccine, Vaccination uptake, Temporal trends, Sex differences, Insurance claims data

## Abstract

**Background:**

Human papillomavirus (HPV) infection can cause various cancers and can be prevented through vaccination. The American Cancer Society (ACS) has set an HPV vaccination completion target in 13-year-old children to 80% by 2026. While HPV vaccine coverage (proportion ever vaccinated) estimates are available, annual uptakes (proportion initiating vaccine in a year) in the United States (U.S.) are not well-known.

**Methods:**

We analyzed MarketScan® claims database to assess HPV vaccination uptakes in the U.S. among the 9- to 26-year-olds in 2006–2016. The annual uptake was the ratio of the number of enrollees who had a first record of an HPV vaccine during the year, and the number of enrollees of similar age and sex that year.

**Results:**

Uptake was below 1% among children turning 9 and 10 years old during the year. Since 2009 among female and since 2013 among males, the annual uptake has been the highest in those turning 13 years old (19.7% among females and 17.6% among males in 2016). Catch-up vaccination among older adolescents and young adults increased after Advisory Committee for Immunization Practices (ACIP) recommendations, but eventually slowed down as more younger persons were vaccinated. Most young adolescents were vaccinated by pediatricians, whereas young adult women were predominantly vaccinated by obstetricians/gynecologists and young adult males by family physicians. While only about half of the adolescents had well-check visits, the majority of those who initiated HPV vaccination had one the same year.

**Conclusion:**

Continued increase in uptake is needed to reach the ACS 2026 goals.

**Supplementary Information:**

The online version contains supplementary material available at 10.1186/s12889-021-11664-1.

## Background

Recent data indicate that approximately 14 million persons are newly infected with human papillomavirus (HPV) each year in the United States (U.S.), and approximately 79 million persons are currently infected [1]. HPV causes most of cervical pre-cancers and cervical cancers, and many vulvar, vaginal, penile, anal, and oropharyngeal cancers, and diseases such as genital warts [2]. In the U.S., a total of 43,371 HPV-associated cancers were reported in 2015 [3]. From an economic perspective, HPV was reported to be the second most expensive sexually transmitted disease after Human Immunodeficiency Virus (HIV) in terms of direct medical care costs [4].

To date, three HPV vaccines have been approved in the U.S. for the prevention of HPV-related diseases: a quadrivalent (4vHPV), a bivalent (2vHPV), and nonavalent (9vHPV) vaccine [5–7]. The 4vHPV was licensed to protect against diseases caused by HPV types 6, 11, 16 and 18, for females in 2006, and for males in 2009 [6]. The 2vHPV vaccine covering types 16 and 18 became available in 2009 for females only [7]. The 4vHPV was the most commonly administered HPV vaccine in the U.S. until the 9vHPV vaccine against HPV types 6, 11, 16, 18, 31, 33, 45, 52, and 58 was approved for use in both males and females in December 2014 [6]. Since the beginning of 2017, 9vHPV has been the only HPV vaccine available in the U.S. [8].

The U.S. Advisory Committee on Immunization Practices (ACIP) recommended routine HPV vaccination of girls at the ages of 11 or 12 in 2006 [9]. Vaccination could start as early as 9 years and catch-up vaccination was recommended through age 26 years for females. In 2011, vaccination was routinely recommended for both girls and boys and catch-up was extended for males through age 21 years [5]. In June 2019, the ACIP harmonized the upper age limit for male and female vaccination catch-up to 26 years, and recommended shared clinical decision-making for adults 27 through 45 years old [10]. Economic models have shown that HPV vaccination of adolescents and young adults is cost-effective, or cost-saving [11]. Recognizing the potential of HPV vaccination in reducing the incidence of vaccine-preventable cancers, the American Cancer Society (ACS) launched an “HPV Cancer Free” public health campaign with a goal to have 80% of 13 year-old girls and boys in the U.S. fully vaccinated with HPV vaccine by 2026, 20 years after introduction of the first HPV vaccine [12]. To meet the ACS goal, it is essential to understand how vaccinations occur. This includes detailed information regarding the age at which children and young adults get vaccinated, who administers the vaccine, and in what setting and visit-type, and the temporal trend around these measures. This information can guide the development of appropriate policy measures and interventions to help meet the ACS goal. Analysis of annual vaccination uptake, i.e. the proportion of persons initiating HPV vaccine in a year, can provide insights on when, where, and by whom females and males get vaccinated. This can be helpful for improving the overall vaccination coverage, which is the proportion of the population who has ever received HPV vaccination during their lifetime. Vaccination coverage has been estimated through insurance claims data [13, 14] and nationwide studies such as the National Health and Nutrition Examination Survey (NHANES) of the 9- to 59-year-olds [15], the National Immunization Survey-Teen (NIS-Teen) among the 13- to 17-year-olds [16], and the National Health Interview Survey (NHIS) targeting males and females aged 19 to 26 years old [17]. None of the studies provided direct estimates of the HPV vaccination uptake. Vaccinations often occur during annual well-check visits, that are recommended by the American Academy of Pediatrics for all U.S. children [18, 19], and understanding how uptake is associated with a well-check visit is also important.

In this study, we assess HPV vaccination uptake since the first HPV vaccine was introduced in 2006 until 2016, within the 9- to 26-year-old population. We also examine who administered the vaccine, and whether vaccines are initiated during a well-check visit.

## Methods

### Data source

This retrospective analysis was based on the IBM® MarketScan® claim database which comprises prescription data in addition to inpatient and outpatient medical utilization for millions of commercially insured individuals across the U.S. In 2016, the database covered approximately 10% of the total U.S. population aged 9 to 26 years old. It was selected because of its size, geographic representativeness, and diversity of plan types. IBM provided the de-identified individual-level data which are fully compliant with U.S. privacy laws and regulations, i.e. the Health Insurance Portability and Accountability Act (HIPAA). As a result, this study was IRB exempt, and no IRB approval was necessary.

### Design, measurements and analysis

The annual HPV vaccination uptake was defined as the proportion of unique enrollees who had a first claim for a dose of the HPV vaccine (Current Procedure Terminology (CPT) code: 90649 (4vHPV), 90651 (9vHPV), and 90650 (2vHPV) during the year. Age of the subject was determined by difference between calendar year and year of birth so that the 1997 newborns were the 9-year-olds in 2006 (the database only provides the year of birth to protect anonymity). Since the HPV vaccine is indicated at 9 years old or above, we could assume, in 2006 for example, that everyone from birth cohort 1997 who was vaccinated was 9 years old. However, in 2006, subjects from birth cohort 1996 could be vaccinated either at 9 or 10 years old depending upon whether they had the vaccine before or after their 10th birthday, and therefore were referred to as the 9–10-year-olds. Each year, females and males turning 9 to 26 years old were included, for example,, in 2006, enrollees who were born between 1980 (turning 26 years old) and 1997 (turning 9 years old). Women with an International Classification of Disease (ICD 9/10) code for pregnancy (Supplementary material, Table S1) recorded anytime during the year were excluded from both the numerator and the denominators of the annual uptakes. A heat map was produced reporting the uptake with red color representing lower uptake, and green color indicating higher uptake. The heat map provides a temporal trend for interpreting uptake over time.

The MarketScan® database also includes data on the type of health care provider that administered the vaccine. Healthcare providers filing the claim were regrouped in various categories; pediatricians, family physicians, neonatal specialists, obstetricians/gynecologists, and primary care physicians based on available categories in MarketScan®. Since there were broad categories, some physician codes were combined and put into “others” category. To assess whether vaccination was initiated during well-check visits, each calendar year, children and adolescents aged 9 to 18 years old who had a medical claim associated with one of the ICD 9/10 diagnoses (primary or secondary) or CPT procedure codes listed in Supplementary material (Table S2) were identified. Those within this group who initiated HPV vaccination were also identified. Data were stratified by calendar year, age (i.e., year of birth) and sex. Analyses were performed using SAS version 9.4, and heat maps depicting uptake were created in Microsoft Excel.

## Results

### HPV vaccination uptake

**Fig. 1:**
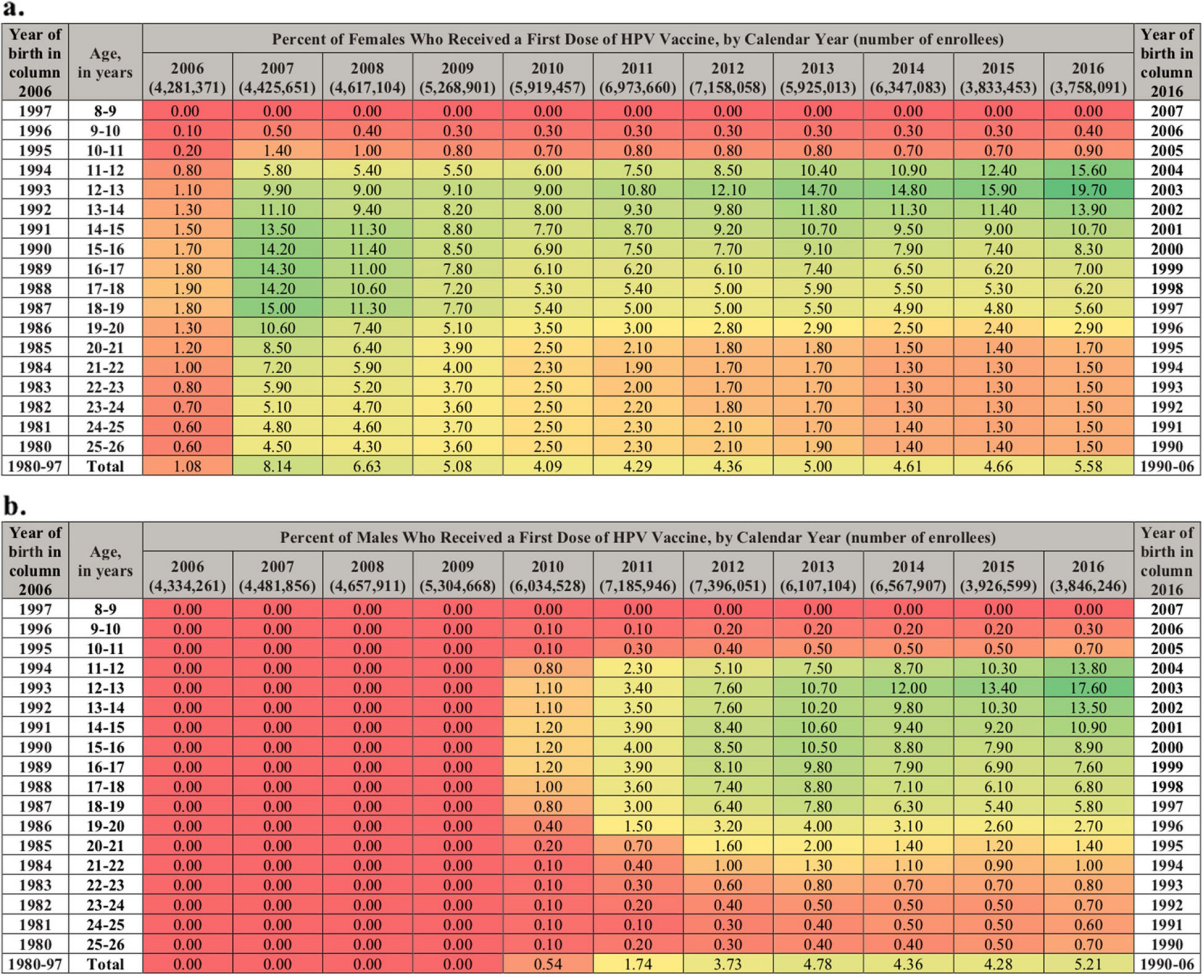
Annual HPV Vaccination Uptake From 2006 to 2016 Among 9- to 26-Year-Olds of the United States: a. Females and b. Males. The annual uptakes are calculated as the percentage of enrollees of same age and sex who had a first claim for an HPV vaccine during the year. The colors in the graph depict the uptake pattern observed over time with colors transitioning from red to green as uptake values get higher. Each year, subjects turning between 9 and 26 years old are included (in 2006, enrollees born between 1980 and 1997, and in 2016, subjects are born between 1990 and 2007). Each column provides the annual uptakes by subjects’ year of age as determined by year of birth (in 2006, the 9–10-year-old subjects are those turning 10 during the year, i.e. the 1996 newborns). Birth cohort annual uptake estimates are found on decreasing diagonals. For example, 0.5 and 5.6% of enrollees born in 1997 initiated HPV vaccination in 2007 and 2016, respectively

In the Marketscan® claims database, there were 118 million unique enrollees who turned 9 to 26 years old from 2006 to 2016. Slightly less than half (49.4%) were females. Figure 1 shows the age-specific annual uptake of HPV vaccination between 2006 and 2016, for females a. and for males b., respectively. Birth cohort-specific uptakes can be found moving diagonally downwards from left to right across the figures. For females who turned 9 years old in 2006, or the 1997 cohort the vaccination uptake was: 1.0% in 2008 (10–11 years old), 9.0% in 2010 (12–13 years old), 9.1% in 2013 (16–17 years old), and 5.6% in 2016 (18–19 years old). Among males born in 1997, the uptake was 0% until HPV vaccination was indicated for males in 2009, after which, we observe catch-up vaccination occurring: 1.1% in 2010 (12–13 years old), 10.5% in 2013 (15–16 years old) and 5.8% in 2016 (18–19 years old). Throughout the study period, the uptake was below 1% among children turning 9 and 10 years old during the year, while it increased gradually in adolescents turning 12 to 14 years old, reaching 19.7% of females and 17.6% of males among those turning 13 years old in 2016. Since 2009 among female and since 2013 among males, the uptake was the highest at 12–13 years old. Among females turning 16 years old and above, the uptake peaked in 2007, when the vaccine became available, then dropped and flattened as cohorts with vaccinated persons aged. Among females turning 22 years old or more, the uptake was low, around 1.5% in 2016. In males, the catch-up among older adolescents spread out more uniformly; The highest uptakes were observed in 2013 (among the 15–16 years old, the annual uptakes from 2011 to 2016 were respectively: 4.0, 8.5, 10.5, 8.8, 7.9 and 8.9%). The uptakes were ≤ 1% among males turning 22 years old or more.

### Well-check visit

The percent of subjects with a documented well-check visit by age and sex for 2016 is reported in Table 1. From year 2006 to 2016, the annual proportion of those turning 9 to 18 years old who had a well-check visit increased from 29.7% (2006) to 49.0% (2016) among females, and from 28.5% (2015) to 47.9% (2016) among males (Table 2). Most of the children and adolescent who initiated HPV vaccine had at least one well-check visit the same year (year- and sex-specific proportions varied between 76.9 and 91.3%, Table 2). The frequency of claims associated with either of the various ICD9/10 diagnosis codes or CPT procedure codes for well-check visits in year 2016 is reported as supplementary material (Table S2).

**Table 1:** Frequency of well-check visits in 2016, by age and sex

Age, in years	Percent of enrollees with a well-check visit in 2016	
	Males	Females
9	48.1	47.2
10	46.9	46.1
11	45.7	45.1
12	53.3	53.8
13	56.7	56.5
14	51.8	50.0
15	48.6	48.4
16	45.8	47.1
17	43.1	47.3
18	40.2	48.3
19	34.2	48.8
20	21.8	43.5
21	15.7	41.5
22	13.8	43.3
23	12.3	43.2
24	11.7	42.7
25	11.9	42.8
26	12.3	43.0

**Table 2:** HPV vaccination uptake and well-check visits among the 9- to 18-years-olds: 2006 to 2016

	Calendar year										
	2006	2007	2008	2009	2010	2011	2012	2013	2014	2015	2016
**Females 9 to 18 years old**											
Total number of enrollees	2,378,859	2,442,856	2,530,358	2,898,580	3,281,162	3,729,837	3,730,210	3,027,639	3,202,509	1,915,796	1,885,722
Number of enrollees who initiated the vaccine series during the year	25,913	214,650	180,162	166,380	166,971	215,305	225,899	218,992	218,879	132,963	157,527
Percent of enrollees who initiated the vaccine series during the year	1.1	8.8	7.1	5.7	5.1	5.8	6.1	7.2	6.8	6.9	8.4
Number of enrollees who had a well-check visit during the year	705,346	786,193	858,370	1,080,995	1,226,767	1,486,044	1,530,606	1,311,181	1,425,307	901,285	923,544
Percent of enrollees who had a well-check visit during the year	29.7	33.2	33.9	37.3	37.4	39.8	41	43.3	44.5	47	49
Number of enrollees who initiated the vaccine series and had a well-check visit during the year	21,137	176,614	144,495	137,383	139,270	184,970	197,813	194,179	193,671	120,103	141,897
Percent of those who initiated the vaccine series during the year who had a well-check visit that year	81.6	82.3	80.2	82.6	83.4	85.9	87.6	88.7	88.5	90.3	90.1
**Males 9 to 18 years old**											
Total number of enrollees	2,481,228	2,548,560	2,638,642	3,023,411	3,426,415	3,886,774	3,882,225	3,151,500	3,335,682	1,993,684	1,960,728
Number of enrollees who initiated the vaccine series during the year	124	744	694	650	26,786	99,984	212,044	221,767	218,544	131,005	159,020
Percent of enrollees who initiated the vaccine series during the year	0	0	0	0	0.8	2.6	5.5	7	6.6	6.6	8.1
Number of enrollees who had a well-check visit during the year	708,350	771,080	842,805	1,074,213	1,238,359	1,501,173	1,555,409	1,335,685	1,449,299	918,200	939,500
Percent of enrollees who had a well-check visit during the year	28.5	30.3	31.9	35.5	36.1	38.6	40.1	42.4	43.4	46.1	47.9
Number of enrollees who initiated the vaccine series and had a well-check visit during the year	103	584	534	537	23,789	88,985	192,487	200,976	197,046	119,667	144,921
Percent of those who initiated the vaccine series during the year who had a well-check visit that year	83.1	78.5	76.9	82.6	88.8	89	90.8	90.6	90.2	91.3	91.1

### Provider type

**Fig. 2:**
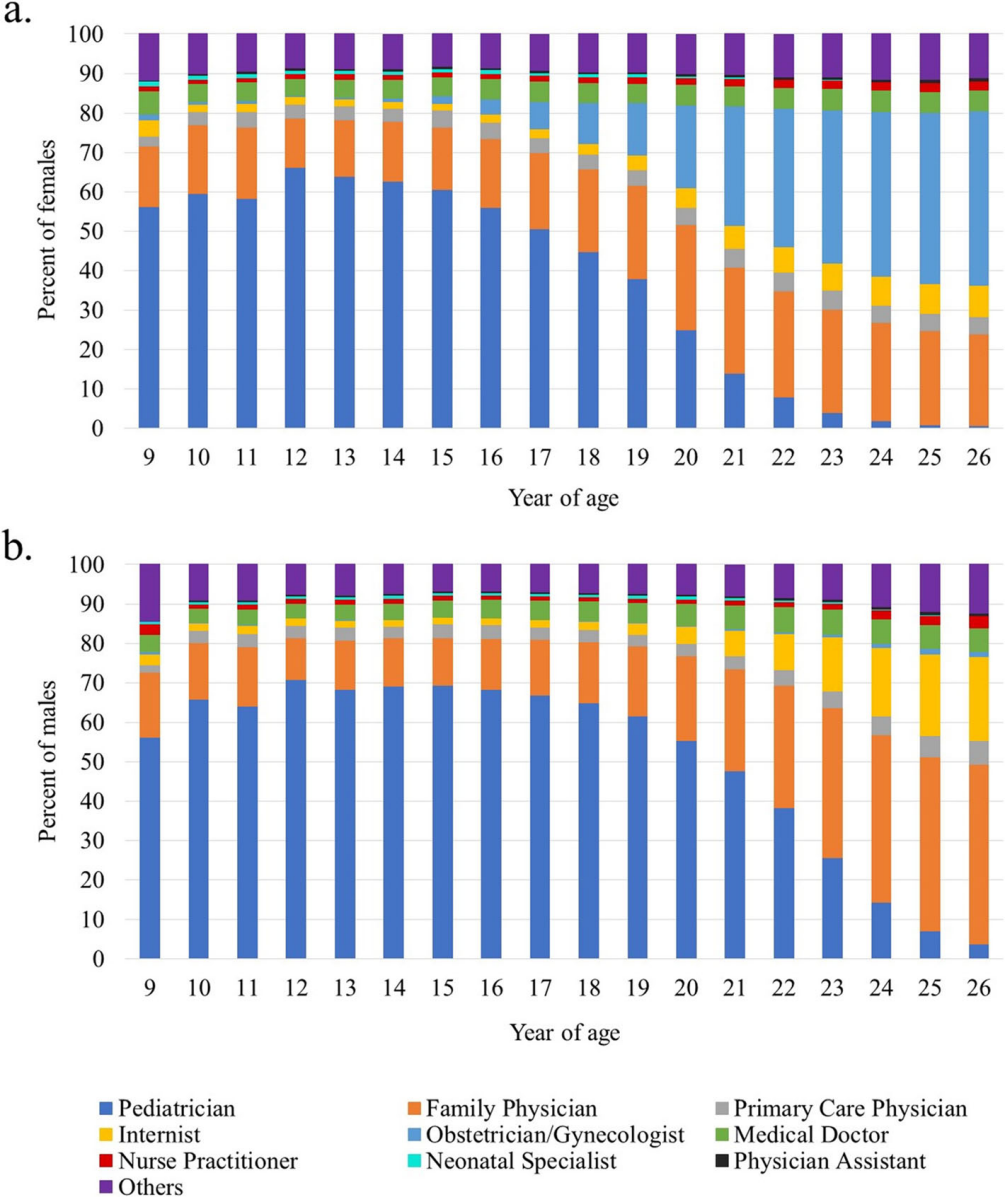
Distribution of Initial HPV Vaccine Dose Providers According to Patient Age and Sex Between 2006 and 2016 Among 9- to 26-Year-Olds of the United States: a. Females and b. Males. Subjects’ year of age was determined by year of birth (e.g. the 9-year-olds are those who turned 9 the year they received their first HPV vaccine)

Figure 2 shows the distribution of health care professionals who provided the initial HPV vaccine dose between 2006 and 2016, according to age and sex of the vaccinated subjects. Pediatricians, family physicians, obstetricians/gynecologists and internists administered the first dose in 60.6, 15.8, 1.8 and 1.7% of girls turning 15 years old, respectively, while among women 26 years old, the respective proportions were 0.6, 23.3, 44.2 and 7.9%. Pediatrician, family physicians, and internists administered the first dose in 69.4, 12.0 and 1.7% of boys turning 15 years old in comparison with 3.7, 45.6 and 21.2% of men turning 26 years old, respectively.

## Discussion

We assessed HPV vaccine uptake in the U.S. within a large claims database covering several million 9 to 26 year-old children and young adults between 2006 and 2016. The study includes all the licensed HPV vaccines [20, 21], and therefore provides a thorough analysis of vaccine uptake in the U.S. and the extent of catch-up implementation during the 10-year period following HPV vaccine licensure (females in mid-2006 and males in late 2009) [22, 23].

Our results seem consistent with another claim-based study by the Blue Cross Blue Shield Association [13], that reported a 32 and 37% vaccine coverage for the 13-year-old male and female adolescents in 2016. In comparison, cumulating the uptakes that we estimated within the birth cohorts turning 13 years old in 2016, the coverage at age 12–13 years old would be approximately 28.6% in males and 33.1% in females. It is also consistent with the numbers from Gargano et al. [14] who used the same underlying MarketScan® database, but who applied a different methodology (in 2014, 53.0% of females and 30.3% of males turning 17 or 18 years old had been vaccinated, versus about 51.7% of females and 30.9% of males cumulating our uptake estimates) using restrictive inclusion criteria requiring more continuous follow-up. While claims-based uptakes from our study seem low as compared to the result from the National Health and Nutrition Examination Survey (NHANES) [15] or from NIS-Teen [16], they cannot be directly compared as they both are vaccination coverage studies that ask for vaccination during the lifetime.

The study provides insight into how a new ACIP recommendation for a vaccine is implemented in a real-world setting. When the HPV vaccine was approved for women in 2006, a broad slice of the population across different age groups got vaccinated. There was catchup vaccination uptake in older age group, with 10.6% of those turning 20 years old and 4.5% of those turning 26 years old receiving a dose of the vaccine. Over time, more and more teenagers were vaccinated at younger ages which means less were available for vaccination as older adolescents and young adults. This translated into higher uptake for young adolescents (14.7 and 18.6% of those turning 12 and 13 years old, respectively) and lower uptake at older ages (1.1% of those turning 26 years old) in 2016. This pattern can be observed through the heat map. As vaccination is initiated, the hue is light green across a broader age range, including older adults. Moving to the right the cells start turning dark green for young adolescents indicating steadily increasing vaccination uptake for this group, whereas it gets lighter for older adolescent and young adults (Fig. 1). This shows that vaccination uptake is steadily increasing for adolescents. The uptakes remain red in those turning 9 or 10 years old, implying very few children receive a vaccine at that age. The uptake rate was particularly low for young children with almost none of those turning 9 years old and less than 0.4% of those turning 10 initiating HPV vaccination in the US—this could be because ACIP does not specifically recommend to routinely vaccinate this age group. Thus, the ACIP recommendation appears more influential than the U.S. FDA label for HPV vaccination. This is understandable as the ACIP recommendation drives coverage and reimbursement of the vaccine. Despite the ACIP recommendation for HPV vaccine initiation at a targeted age of 11 or 12 years old (or before), of these turning 12 years of age in 2016, only 1 in 6 girls and 1 in 7 boys received an HPV vaccination, while half of them had a well-check visit. Using our 2016 uptake estimates, the one-dose HPV vaccination coverage of the 13-year-old girls and boys could be approximately 40%. In order to reach the ACS vaccination completion target of 80% by 2026 among 13-year-olds [12], it will be necessary that the annual uptakes in young adolescents continue to increase markedly each year.

The proportion of children and teenagers having a well-check visit increased gradually for both females and males. Our estimates are consistent with previously reported proportions of well-check visits in national surveys for these ages [24–26] and similar to estimates of adolescents and young adults aged 12 to 21 years old who had commercially insured well-care visits each year between 2006 and 2016 [27]. Almost half of the young adolescent population had a well-check visit. Visits tapered for both men and women, and more drastically for men. This is consistent with the literature that shows that women have better health-seeking behaviors [28]. Low well-check rates is a lost opportunity for vaccination as these visits constitute one of the best routes for initiating or completing vaccination series—more than 90% of those who initiated a vaccination series in 2016 also had a well-check visit that year.

Our study also shows that the type of provider for HPV vaccine changes as children age. Pediatricians administer most of the HPV vaccination for children, followed by family physicians. As children grow, their visits to pediatricians decline. In young adulthood, HPV vaccination is mostly picked up by obstetrician/gynecologists for women, and by family physicians and other primary care physicians for men. Among both females and males, a very small proportion had their initial HPV vaccine administered by a nurse practitioner or a physician assistant. These data are important as healthcare providers who administer the vaccine are known to address parental safety concerns, and help promote positive beliefs about vaccination; physician recommendation often is identified as among the most important determinants of HPV vaccination coverage [29, 30]. Our description of who initiates the HPV vaccine series, and in what setting can also be very helpful for implementing interventions aimed at maintaining and improving overall vaccination coverage in specific target populations. For example, well-check visits are recommended once a year in adolescents children and interventions targeted to improve the rate of well-check visits in pediatricians’ office may help increase HPV vaccination uptake in this age group [29, 31]. Interventions that improve well-check visits in older adults can help improve vaccination uptakes in those populations.

Several limitations apply to this study including those inherent to administrative claims database analyses such as misclassification, upcoding and under coding. The study population is also limited to a commercially insured population which may not allow for generalizing the results to the entire U.S. For example, uptakes potentially vary according to ethnicity and socio-economic status, and access to care may differ in the overall population, especially those getting vaccinated under the Vaccines for Children (VFC) program. We also could not identify vaccines not submitted for insurance reimbursement nor those that were received out-of-network as for example, enrollees were not all continuously in the database since 2006. Only non-missing values were considered, and no imputation of missing information was carried out. Lastly, only birth year was available so vaccine coverage by exact year of age could not be determined.

## Conclusions

We found that the ACIP vaccine recommendations directly impact HPV vaccination uptake in the population. When the HPV vaccination program started in 2006 for females and 2009 for males, there was catch-up in a broader age-group, followed by an increase in vaccine uptake among targeted young cohorts. While females had a head start (with early inclusion in the label for HPV vaccination) vaccination uptake in males is fast catching up. While uptake in young adolescents has been increasing, coverage is far below the ACS 80% goal, and catch-up vaccination in older cohorts is likely to continue into the near future as many children remain unvaccinated as they get older. Pediatricians, followed by family physicians remain the primary vaccine administrators for adolescents. Obstetrician/gynecologists and family physicians commonly vaccinate young adult women and young adult men, respectively. Thus, interventions to improve uptake should consider focusing on pediatricians and family physicians for children, and obstetrician/gynecologists and family physicians for young adults. Interventions to improve access to well-check visits and HPV vaccination within well-check visits should also be considered to improve HPV uptake. Our study provides an analysis of how many children and young adults get vaccinated, when, by whom, and in what context. This can guide the development of appropriate interventions and policies to help improve vaccination uptake and public health.

## Supplementary Information

**Additional file 1: Table S1.** ICD-9/10 codes used to identify pregnancy-related service claims. **Table S2.** ICD9/10 and CPT codes used to define well-check visits.

## Data Availability

The study is based on de-identified individual-level data from a secondary administrative healthcare claims database (IBM® MarketScan® Research Database) which are fully compliant with U.S. privacy laws and regulations, i.e. the Health Insurance Portability and Accountability Act (HIPAA). The data that support the findings of this study are available from IBM® MarketScan® Research Databases, but restrictions apply to the availability of these data, which were used under license for the current study, and so are not publicly available. Data are however available from the authors upon reasonable request and with permission of IBM® Watson Health™.

## Abbreviations

HPV: Human papillomavirus
US: United States
ACIP: Advisory Committee for Immunization Practices
HIV: After Human Immunodeficiency Virus
4vHPV: Quadrivalent HPV vaccine
2vHPV: Bivalent HPV vaccine
9vHPV: Nonavalent HPV vaccine
ACS: American Cancer Society
NHANES: National Health and Nutrition Examination Survey
NIS-Teen: National Immunization Survey-Teen
NHIS: National Health Interview Survey
CPT: Current Procedure Terminology
ICD: An International Classification of Disease
VFC: Vaccines for Children
