# Supplementary material for: HPV vaccination uptake and administration from 2006 to 2016 in a commercially insured population of the United States
Source: BMC Public Health. 2021 Sep 6;21:1629. doi: 10.1186/s12889-021-11664-1 (PMC8422649; doi:10.1186/s12889-021-11664-1)
Supplement: Supplementary file 1 — Additional file 1: Table S1. ICD-9/10 codes used to identify pregnancy-related service claims. Table S2. ICD9/10 and CPT codes used to define well-check visits. [file 12889_2021_11664_MOESM1_ESM.docx]

Table S1: ICD-9/10 codes used to identify pregnancy-related service claims

| **Code** | **Description** |
| --- | --- |
| 640.x-649.x | Complications mainly related to pregnancy |
| 650.x-659.x | Normal delivery, and other indications for care in pregnancy, labor, and delivery |
| 660.x-669.x | Complications occurring mainly in the course of labor and delivery |
| V22.x | [Normal pregnancy](http://icd9cm.chrisendres.com/index.php?action=child&recordid=10788) |
| V23.x | Supervision of high-risk pregnancy |
| V27.x | Outcome of delivery |
| V28.x | Encounter for antenatal screening of mother |
| V72.42 | Pregnancy examination or test, positive result |
| Z34.x | Encounter for supervision of normal pregnancy |
| [O60-O77](http://www.icd10data.com/ICD10CM/Codes/O00-O9A/O60-O77) | Complications of labor and delivery |
| [O80-O82](http://www.icd10data.com/ICD10CM/Codes/O00-O9A/O80-O82) | Encounter for delivery |
| O60-O77 | Complications of labor and delivery |
| O00-O09 | Pregnancy with abortive outcome |

Table S2: ICD9/10 and CPT codes used to define well-check visits

| **Code** | **Description** | **Frequency in 2016** |
| --- | --- | --- |
| Z00129 | Encounter for routine child health examination without abnormal findings | 1,711,459 |
| Z0000 | Encounter for general adult medical examination without abnormal findings | 669,502 |
| 99394 | Periodic comprehensive preventive medicine reevaluation and management of an individual including an age and gender appropriate history, examination, counseling/anticipatory guidance/risk factor reduction interventions, and the ordering of laboratory/diagnostic procedures, established patient; adolescent (age 12 through 17 years) | 541,582 |
| Z01419 | Encounter for gynecological examination (general) (routine) without abnormal findings | 443,303 |
| 99393 | Periodic comprehensive preventive medicine reevaluation and management of an individual including an age and gender appropriate history, examination, counseling/anticipatory guidance/risk factor reduction interventions, and the ordering of laboratory/diagnostic procedures, established patient; late childhood (age 5 through 11 years) | 393,695 |
| 99395 | Periodic comprehensive preventive medicine reevaluation and management of an individual including an age and gender appropriate history, examination, counseling/anticipatory guidance/risk factor reduction interventions, and the ordering of laboratory/diagnostic procedures,18-39 years | 362,165 |
| 99385 | Initial comprehensive preventive medicine evaluation and management of an individual including an age and gender appropriate history, examination, counseling/anticipatory guidance/risk factor reduction interventions, and the ordering of laboratory/diagnostic procedures, new patient;18-39 years | 124,067 |
| Z3009 | Encounter for other general counseling and advice on contraception | 84,799 |
| Z3041 | Encounter for surveillance of contraceptive pills | 75,654 |
| Z3042 | Encounter for surveillance of injectable contraceptive | 65,605 |
| 99384 | Initial comprehensive preventive medicine evaluation and management of an individual including an age and gender appropriate history, examination, counseling/anticipatory guidance/risk factor reduction interventions, and the ordering of laboratory/diagnostic procedures, new patient;adolescent (age 12 through 17 years) | 55,242 |
| Z30011 | Encounter for initial prescription of contraceptive pills | 46,348 |
| Z025 | Encounter for examination for participation in sport | 45,830 |
| Z3049 | Encounter for surveillance of other contraceptives | 42,563 |
| 99383 | Initial comprehensive preventive medicine evaluation and management of an individual including an age and gender appropriate history, examination, counseling/anticipatory guidance/risk factor reduction interventions, and the ordering of laboratory/diagnostic procedures, new patient; late childhood (age 5 through 11 years) | 32,330 |
| Z3040 | Encounter for surveillance of contraceptives, unspecified | 29,984 |
| 99420 | Administration and interpretation of health risk assessment instrument, e.g., health hazard appraisal | 26,839 |
| Z0289 | Encounter for other administrative examinations | 22,498 |
| 99401 | Preventive medicine counseling and/or risk factor reduction intervention(s) provided to an individual (separate procedure); approximately 15 minutes | 22,300 |
| Z008 | Encounter for other general examination | 20,047 |
| Z30018 | Encounter for initial prescription of other contraceptives | 13,725 |
| Z029 | Encounter for administrative examinations, unspecified | 13,312 |
| Z020 | Encounter for examination for admission to educational institution | 12,955 |
| Z30013 | Encounter for initial prescription of injectable contraceptive | 12,417 |
| 99402 | Preventive medicine counseling and/or risk factor reduction intervention(s) provided to an individual (separate procedure); approximately 30 minutes | 9,326 |
| Q0091 | Screening papanicolaou smear; obtaining, preparing and conveyance of cervical or vaginal smear to laboratory | 8,653 |
| S0612 | Annual gynecological examination, established patient | 6,495 |
| Z003 | Encounter for examination for adolescent development state | 6,421 |
| Z021 | Encounter for pre-employment examination | 5,068 |
| Z30019 | Encounter for initial prescription of contraceptives, unspecified | 4,118 |
| Z30014 | Encounter for initial prescription of intrauterine contraceptive device | 3,564 |
| Z3002 | Counseling and instruction in natural family planning to avoid pregnancy | 2,958 |
| G0101 | Cervical or vaginal cancer screening; pelvic and clinical breast examination | 2,246 |
| S0610 | Annual gynecological examination, new patient | 2,149 |
| Z0279 | Encounter for issue of other medical certificate | 2,147 |
| 99404 | Preventive medicine counseling and/or risk factor reduction intervention(s) provided to an individual (separate procedure); approximately 60 minutes | 2,128 |
| 99403 | Preventive medicine counseling and/or risk factor reduction intervention(s) provided to an individual (separate procedure); approximately 45 minutes | 1,487 |
| Z024 | Encounter for examination for driving license | 728 |
| 99391 | Periodic comprehensive preventive medicine reevaluation and management of an individual including an age and gender appropriate history, examination, counseling/anticipatory guidance/risk factor reduction interventions, and the ordering of laboratory/diagnostic procedures, established patient; infant (age younger than 1 year) | 721 |
| 99429 | Other Preventive Medicine Services | 685 |
| Z0282 | Encounter for adoption services | 463 |
| G0438 | Annual wellness visit, includes a personalized prevention plan of service (PPPS), first visit | 431 |
| 99381 | Initial comprehensive preventive medicine evaluation and management of an individual including an age and gender appropriate history, examination, counseling/anticipatory guidance/risk factor reduction interventions, and the ordering of laboratory/diagnostic procedures, new patient; infant (age younger than 1 year) | 391 |
| G0439 | Annual wellness visit, includes a personalized prevention plan of service (PPPS), subsequent visit | 371 |
| Z022 | Encounter for examination for admission to residential institution | 333 |
| Z026 | Encounter for examination for insurance purposes | 327 |
| 99396 | Periodic comprehensive preventive medicine reevaluation and management of an individual including an age and gender appropriate history, examination, counseling/anticipatory guidance/risk factor reduction interventions, and the ordering of laboratory/diagnostic procedures,40-64 years | 276 |
| V202 | Routine infant or child health check | 253 |
| Z023 | Encounter for examination for recruitment to armed forces | 222 |
| 99392 | Periodic comprehensive preventive medicine reevaluation and management of an individual including an age and gender appropriate history, examination, counseling/anticipatory guidance/risk factor reduction interventions, and the ordering of laboratory/diagnostic procedures, established patient;early childhood (age 1 through 4 years) | 174 |
| S0613 | Annual gynecological examination; clinical breast examination without pelvic evaluation | 145 |
| G0402 | Initial preventive physical examination; face-to-face visit, services limited to new beneficiary during the first 12 months of medicare enrollment | 132 |
| V700 | Routine general medical examination at a health care facility | 81 |
| Z0271 | Encounter for disability determination | 81 |
| 99386 | Initial comprehensive preventive medicine evaluation and management of an individual including an age and gender appropriate history, examination, counseling/anticipatory guidance/risk factor reduction interventions, and the ordering of laboratory/diagnostic procedures, new patient;40-64 years | 67 |
| V7231 | Routine gynecological examination | 49 |
| 99382 | Initial comprehensive preventive medicine evaluation and management of an individual including an age and gender appropriate history, examination, counseling/anticipatory guidance/risk factor reduction interventions, and the ordering of laboratory/diagnostic procedures, new patient;early childhood (age 1 through 4 years) | 33 |
| S0622 | Physical exam for college, new or established patient (list separately in addition to appropriate evaluation and management code | 23 |
| Z0281 | Encounter for paternity testing | 18 |
| 99397 | Periodic comprehensive preventive medicine reevaluation and management of an individual including an age and gender appropriate history, examination, counseling/anticipatory guidance/risk factor reduction interventions, and the ordering of laboratory/diagnostic procedures, established patient;65 years and older | 13 |
| V2549 | Surveillance of other contraceptive method | 8 |
| V2541 | Surveillance of contraceptive pill | 6 |
| V2502 | General counseling on initiation of other contraceptive measures | 5 |
| 99387 | Initial comprehensive preventive medicine evaluation and management of an individual including an age and gender appropriate history, examination, counseling/anticipatory guidance/risk factor reduction interventions, and the ordering of laboratory/diagnostic procedures, new patient;65 years and older | 4 |
| G0468 | Federally qualified health center (fqhc) visit, ippe or awv; a fqhc visit that includes an initial preventive physical examination (ippe) or annual wellness visit (awv) and includes a typical bundle of medicare-covered services that would be furnished per diem to a patient receiving an ippe or awv | 3 |
| V2509 | Other general counseling and advice on contraceptive management | 3 |
| V2501 | General counseling on prescription of oral contraceptives | 2 |
| V2540 | Contraceptive surveillance, unspecified | 2 |
| V2543 | Surveillance of implantable subdermal contraceptive | 2 |
| V703 | Other general medical examination for administrative purposes | 1 |
| V705 | Health examination of defined subpopulations | 1 |
| V212 | Other development of adolescence | 0 |
| V250 | General counseling and advice on contraceptive management | 0 |
| V2504 | Counseling and instruction in natural family planning to avoid pregnancy | 0 |
| V254 | Surveillance of previously prescribed contraceptive methods | 0 |
| V2542 | Surveillance of intrauterine contraceptive device | 0 |
| V709 | Unspecified general medical examination | 0 |
| V723 | Gynecological examination | 0 |
| Z000 | Encounter for general adult medical examination without abnormal findings | 0 |
| Z0012 | Encounter for routine child health examination | 0 |
| Z014 | Encounter for gynecological examination | 0 |
| Z0141 | Encounter for routine gynecological examination | 0 |
| Z02 | Encounter for administrative examination | 0 |
| Z027 | Encounter for issue of medical certificate | 0 |
| Z028 | Encounter for other administrative examinations | 0 |
| Z300 | Encounter for general counseling and advice on contraception | 0 |
| Z3001 | Encounter for initial prescription of contraceptives | 0 |
| Z30015 | Encounter for initial prescription of vaginal ring hormonal contraceptive | 0 |
| Z30016 | Encounter for initial prescription of transdermal patch hormonal contraceptive device | 0 |
| Z30017 | Encounter for initial prescription of implantable subdermal contraceptive | 0 |
| Z304 | Encounter for surveillance of contraceptives | 0 |
| Z3043 | Encounter for surveillance of intrauterine contraceptive device | 0 |
| Z3044 | Encounter for surveillance of vaginal ring hormonal contraceptive device | 0 |
| Z3045 | Encounter for surveillance of transdermal patch hormonal contraceptive device | 0 |
| Z3046 | Encounter for surveillance of implantable subdermal contraceptive | 0 |
| V706 | Health examination in population surveys | 0 |
| V708 | General medical exam nec (other specified general medical examinations) | 0 |
| G0344 | Initial Preventive Physical Examination; Face-To-Face Visit, Services Limited | 0 |
